# ‘There has to be some chemistry there’: an interpretive description exploring the experiences, motivations and dynamics of partnered child health research

**DOI:** 10.1186/s40900-025-00777-1

**Published:** 2025-08-29

**Authors:** Leah K. Crockett, S. Michelle Driedger, Shannon D. Scott, Kent C. Loftsgard, Kathryn M. Sibley

**Affiliations:** 1 Department of Pediatrics and Child Health, University of Manitoba, 532-715 McDermot Avenue, Winnipeg, R3E 3P4 MB Canada; 2 Children’s Hospital Research Institute of Manitoba, Winnipeg, MB Canada; 3 College of Community and Global Health, Rady Faculty of Health Sciences, University of Manitoba, S113-750 Bannatyne Avenue, Winnipeg, R3E 0W3 MB Canada; 4 Faculty of Nursing, University of Alberta, 11405 87 Avenue, Edmonton, T6G 1C9 AB Canada; 5 CIHR Strategy for Patient Oriented Research, #106 -105 2nd St. West, Vancouver, V7M 0E3 BC Canada; 6 George & Fay Yee Centre for Healthcare Innovation, 379-753 McDermot Avenue, Winnipeg, R3E 0W3 MB Canada

**Keywords:** Research partnerships, Integrated knowledge translation, Community-based participatory research, Action research, Patient-oriented research

## Abstract

**Background:**

Research partnerships between researchers and knowledge users are increasingly valued for their role in generating relevant and impactful research. However, the dynamics of these partnerships, including motivations, challenges, and relational aspects, remain underexplored. This study aimed to understand the experiences of researchers and knowledge users engaged in partnered child health research.

**Methods:**

We used an interpretive description approach to conduct and analyze semi-structured interviews with researchers and knowledge users involved in Canadian partnered child health research projects. Data analysis was iterative, involving inductive coding, theme development, and reflection with an interprofessional colleague with lived experience of a congenital health condition.

**Results:**

Fifteen individuals (12 researchers and 3 knowledge users) participated. We identified three key themes. (1) *Diverse drivers: role-based motivations and perceived effects* – Participants described diverse, role-based motivations for engaging in partnered research. Researchers were primarily driven to enhance research impact, while knowledge users emphasized advocacy and credibility. Individuals embodying dual roles with research expertise and professional experience focused on practical relevance and improving health outcomes. (2) *It’s all about the relationships* – Relational quality was central to successful partnerships. While power dynamics shifted towards greater equity over time, achieving fully equitable relationships remained complex. (3) *Navigating evolutions and tensions* – Academic structures continue to create tensions despite a growing cultural shift toward embracing partnership approaches. Key challenges included insufficient recognition for partnership efforts, and funding mechanisms often misaligning with partnership principles.

**Conclusions:**

This study underscores the importance of understanding role-specific motivations, prioritizing relational quality, and addressing systemic challenges in child health research partnerships. Findings provide insights into key dynamics that help navigate both successes and challenges in these partnerships. Continued efforts are needed to address misalignments across systems and structures, support relationship building, and promote sustained collaboration.

**Supplementary Information:**

The online version contains supplementary material available at 10.1186/s40900-025-00777-1.

## Abstract (plain language summary)

In health research, researchers, patients, families, and healthcare professionals work together more often to ensure that results make a difference in the real world. We know that these partnerships have become more common, but we do not know why people work together, their challenges, and what makes partnerships successful. To learn more, we interviewed 15 Canadian researchers and people involved in child health research partnerships. A colleague living with a congenital health condition collaborated with us to analyze the data and explain the findings. We learned three main things. First, people were motivated to partner for different reasons. Researchers wanted to make their work more useful, while knowledge users often wanted to advocate for change. Some participants had research skills and professional experiences, giving them a unique view of practical research with tangible outcomes. Second, university and government funding systems have not adapted to the needs of researchers and knowledge users working together. For example, these projects require significant time and effort, but working together is not always recognized by universities. Finally, strong relationships are central to working together. Trust, respect, and fairness are the foundation, but these relationships take time to develop. Balancing power between researchers and knowledge users is another challenge. This study highlights the importance of understanding people’s motivations for engaging in research partnerships, improving how they are supported, and prioritizing strong, respectful relationships.

**Supplementary Information:**

The online version contains supplementary material available at 10.1186/s40900-025-00777-1.

## Introduction

Research partnerships, which bring together academic researchers and knowledge users such as healthcare providers, and policymakers, and parents throughout the research process, are increasingly recognized as a pathway for enhancing the relevance and uptake of health research [1, 2]. These partnerships foster the co-production of knowledge by integrating diverse perspectives and aligning research goals with real-world needs [3]. The term research partnerships serves as an umbrella term encompassing a range of collaborative approaches, including integrated knowledge translation (iKT), community-based participatory research (CBPR), and patient engagement (PE) [4]. While these traditions differ in primary focus and origin, they share a commitment to collaboration, reciprocity, and incorporating diverse forms of knowledge [5] while tailoring methods to context and purpose [6].

In child health, research partnerships play a particularly crucial role due to the unique needs of children, who often depend on a range of personal networks and professional services to support their development [7]. In this study, child health refers to the health and well-being of individuals from birth to 18 years, encompassing physical, psychological, and preventative aspects of health [8]. Partnerships in child health may involve engaging directly and indirectly with a range of individuals, each playing a role in shaping child health research [9]. Consequently, partnered child health research often requires coordination across sectors and disciplines [10, 11] and emphasizes the interconnected well-being of children and their families [12]. Partnering with relevant child health knowledge users helps ensure that research is responsive to the needs of children and families and supports its practical application [13].

Despite their potential, research partnerships across all areas of health face well-documented challenges such as competing priorities, institutional barriers, and power dynamics [14, 15]. The systems where partnerships operate, such as academic and healthcare structures, may also create obstacles to inclusive and equitable collaboration [16]. These challenges may be exacerbated in child health research, where ethical considerations, family roles and responsibilities, and the unique challenges of working with children and their families introduce additional complexity [17, 18].

Importantly, motivations for engaging in partnership work, and the relationships that develop through it, are central to how partnerships function [19–21]. Yet, these interpersonal dimensions are often underexplored in the literature. Existing research has predominantly explored broad partnership characteristics, practices, and effects [22–24], or has been project- or network-specific [25, 26], leaving gaps in understanding the mechanisms, motivations, and relational dynamics that influence partnership functioning [23, 26, 27]. Trust is a particularly important dimension of this work [20, 28], not only as a facilitator, but because research itself carries a legacy of harm, particularly for historically underserved populations [29]. As such, the position and trustworthiness of the researcher are not neutral and can influence how partnerships are initiated and sustained [29, 30]. Addressing these gaps is important for refining theoretical and practical insights into how partnerships evolve, respond to challenges, and achieve their goals [31]. Insights from diverse partnership experiences can inform strategies to navigate common challenges and enhance collaborative research efforts.

A qualitative approach is well-suited for examining these dynamics, offering deeper insights into how researchers and knowledge users experience and navigate partnership work. This includes exploring how teams navigate power dynamics, collaborative structures, and relational complexities [29], which may be particularly pronounced in child health research due to the diverse roles of partners, heightened ethical considerations, and the need for research to drive tangible benefits for children and families [9, 13, 18, 32]. By unpacking these experiences, we can gain valuable insights into the realities of conducting partnered health research in child health and beyond.

### Study aim

This study aimed to understand the experiences and perspectives of researchers and knowledge users engaged in partnered child health research. Specifically, we explored what motivates individuals to engage in partnered health research, how these motivations influence the partnership process and its effects, and how researchers and knowledge users navigate partnerships, including factors that contribute to success and challenges.

## Methodology and methods

### Context and study design

This study was conducted as part of the lead author’s (LKC) doctoral research on partnered child health research. We adopted a pragmatic approach [33], focusing on the potential utility of our findings for partnered health research practice. Pragmatism was appropriate given the applied nature of the topic and the diversity of participant roles and perspectives, allowing for flexibility in study design and an emphasis on producing insights relevant to practice. Consistent with this orientation, we used an exploratory approach informed by interpretive description, a qualitative methodology that extends beyond description to understand the underlying meanings and contexts shaping individual experiences, particularly in applied health settings [34].

The research team brought a range of lived, learned, and professional expertise from Canada. The lead author (LKC), a doctoral trainee at the time, conducted all interviews and led analysis. Her background includes knowledge translation, implementation science, and partnered research in pediatric settings. She maintained a reflexive journal and analytic memos throughout the study, with a focus on how her own assumptions and position, as someone who has worked across academic and non-academic roles, might influence interpretation. These reflections were discussed with the academic advisory team and a knowledge user collaborator (KCL) to support analytic depth and reflexivity. KCL’s external, intersecting, personal, and professional perspectives in curriculum design and delivery as a team-based clinical educator, at multiple universities and partnered health research competencies trainer and pediatric neurology research collaborator with funded research networks and research institutes are complemented by his lived experience of congenital cerebral palsy and frequent childhood hospitalizations due to chronic pediatric asthma. LKC and KCL were initially introduced as a result of the preceding research partnership between KCL and KMS since 2017. KCL contributed data analysis, interpretation, and manuscript preparation, offering a rare and valuable blend of skills and perspectives to the study. Engagement with KCL was remotely facilitated by virtual meetings and emails, aligning with the *Involve* level on the IAP2 Spectrum of Public Participation [35]. The academic team (KMS, SMD, SDS) are all professors in health disciplines in Canadian research-intensive universities who practice and study partnered health research across child, Indigenous, and rehabilitation health research contexts. They served in an advisory and mentorship role to the lead author throughout the study. All authors share a commitment to advancing the science and practice of partnered health research. Because this study explored interview participants’ experiences across multiple partnered research projects, no single model or framework guided the partnerships described. Similarly, no framework guided the involvement of KCL whose contributions were shaped by shared interest and collaboratively negotiated based on availability and preference.

We report involvement using the Guidance for Reporting Involvement of Patients and the Public (GRIPP2) short form checklist [36] (supplementary file 1), and methods and findings following the Consolidated Criteria for Reporting Qualitative Research (COREQ) checklist [37].

### Study population and sampling

Participants were drawn from a national cross-sectional survey of Canadian partnered health research projects funded between 2011 and 2019 [38]. In the survey, respondents self-identified as researchers or knowledge users based on the Canadian Institute of Health Research (CIHR)-provided definitions [39]. CIHR defines knowledge users as “individuals, groups, or organizations who may be able to use research to make decisions about health policies, programs, or practices” [39]. As Canada’s federal health research funding agency, CIHR plays a central role in shaping expectations for partnered research. Its definitions provide a shared language for how roles are conceptualized and operationalized in funding applications and reporting. We reviewed survey responses and associated project titles and abstracts to identify those that met our predefined child health criteria. Respondents who met these criteria and agreed to future contact comprised our sampling frame.

### Recruitment

We used multiple sampling approaches, conducted in three phases to invite all 44 eligible individuals: (1) *maximum variation sampling* (July-October 2021): invited participants from completed projects (*n* = 12) and all respondents who self-identified as knowledge users (*n* = 5) ; (2) *targeted sampling* (October 2021): invited individuals to diversify gender, career stage, and content areas (*n* = 8) and (3) *convenience sampling*: invited all remaining eligible individuals (*n* = 19). Invitations were emailed using contact information provided in the survey.

### Data collection

LKC developed a semi-structured interview guide (supplementary file 2), pilot-tested for clarity and content with one researcher and one knowledge user, and led data collection. One pilot interview was not included in the analysis, as the participant had not previously participated in the survey. The other pilot interview met all inclusion criteria and was included in the analysis due to the richness of experience. This individual also contributed to the study as a co-author. This overlap was considered as part of a reflexive approach to analysis. The guide focused on participants’ overall experiences with partnered research, including their motivations for engagement, partnership roles, and challenges and successes encountered. Specific questions probed decision-making processes, power dynamics, and the strategies employed across partnerships and with various knowledge users. The interview guide was informed by Jull et al.’s conceptual framework for knowledge user engagement [40], which guided the inclusion of questions addressing the domains of ethical principles, values, and relational processes. Interviews were conducted via Zoom or telephone, based on participant preference, and were audio-recorded. A reflective journal was maintained to document observations, serving as an additional data source.

### Data analysis

LKC led data analysis and followed an iterative process consistent with interpretive description [34]. Data collection and analysis occurred concurrently. Interviews were transcribed verbatim by an independent service (Transcript Heroes), reviewed for accuracy, and anonymized. Coding was performed inductively using NVivo 12, beginning with initial codes that captured surface-level content described by participants. KCL independently reviewed two transcripts and discussed reflections with LKC, offering contextual insights informed by personal experiences in healthcare and health research. A codebook was then developed to define codes and refine those with overlapping conceptualizations. This codebook was continuously refined through ongoing discussions with KMS. Patterns and themes were identified through constant comparison and repeated engagement with the data [34]. Initial themes were drafted by LKC and further refined through discussion with all team members. Data querying facilitated the identification of patterns and variations in how participants discussed interpretive and thematic ideas, enabling comparisons across cases and by participant attributes (e.g., roles). Feedback from SMD, SDS and multiple iterations of written drafts further refined the analysis. An audit trail was maintained throughout to ensure analytic rigour and transparency.

### Ethics approval

This study received ethics approval from the University of Manitoba Health Research Ethics Board (HS24838/H2021:224). Participants were offered a $25 Visa gift card. Participant vouchers were not contingent on interview completion, and no participants withdrew after beginning the interview. A small number of participants declined the honorarium.

## Results

### Participants

Forty-four individuals were invited to participate (*n* = 39 researchers, 5 knowledge users). Of these, seventeen interviews were scheduled, and fifteen were completed, with interview duration ranging from 25 to 59 min (mean: 40 min). Two individuals cancelled and could not be rescheduled, three declined due to workload, and three expressed interest but were unavailable despite follow-up. The remaining invitees did not respond. Most interviews were conducted via Zoom (*n* = 14), with one by telephone.

### Duals: having a foot in both worlds

Participants were initially categorized based on survey self-identification as researchers (*n* = 12) or knowledge users (*n* = 3). While these designations informed sampling and recruitment, through analysis, we found that 5 participants initially categorized as researchers also had relevant health professional training, work experience, or decision-making authority relevant to child health. These individuals straddled academic and healthcare settings, a positioning we referred to as individuals with dual researcher and knowledge user experiences (hereafter referred to as duals for brevity). While we use this label as an analytic shorthand, it does not represent a self-defined identity, nor does it suggest a singular or fixed social position. Further conceptual reflections on dual roles are discussed in the Discussion section, including connections to the literature on boundary spanners, embedded researchers, and outsider-within perspectives. Duals combined the analytical skills of researchers with the practical, applied perspectives of knowledge users. Throughout the interviews, duals sometimes had to clarify their roles, underscoring the fluid and sometimes ambiguous nature of their positioning. Our analysis considers the perspectives of 5 duals, 7 researchers, and 3 knowledge users.

### Participant characteristics

Table 1 summarizes participant attributes. To protect participant anonymity, we do not display by role grouping. Most interview participants identified as female (*n* = 12, 80%) and were based in several Canadian provinces, with Ontario most represented (*n* = 4, 29%). Participants worked across diverse areas of child health, with the most common focus being health systems and services (*n* = 6, 40%). Researchers and duals were evenly distributed between mid-career (5 to 15 years) (*n* = 6, 50%) and senior career (greater than 15 years) stages (*n* = 6, 50%). One participant self-identified as Indigenous (*n* = 6.7%) and one as a visible minority (*n* = 6.7%). Three individuals contributed knowledge user perspectives: a person with lived experience of a health condition, a manger or administrator, and a representative from a healthcare professional organization. For dual-role participants, a knowledge user lens was inferred based on their professional affiliation described during the interview, most commonly healthcare professionals in addition to their primary role as an academic researcher.

**Table 1:** Participant characteristics

Characteristic	n (%)
***Research partnership role***	
Researcher	7 (46.7)
Dual	5 (33.3)
Knowledge user	3 (20.0)
***Province***	
Ontario	4 (26.6)
Manitoba	3 (20.0)
Alberta	3 (20.0)
British Columbia	3 (20.0)
Quebec	1 (6.7)
Nova Scotia	1 (6.7)
***Research pillar***	
Health systems and services	6 (40.0)
Social, cultural, environmental and population health	4 (26.7)
Clinical	3 (20.0)
Biomedical	0 (0.0)
N/A	2 (13.3)
***Gender***	
Female	12 (80.0)
Male	3 (20.0)
***Self-identifies as a visible minority***	
Yes	1 (6.7)
No	14 (92.3)
***Self-identifies as Indigenous***	
Yes	1 (6.7)
No	14 (92.3)
***Academic Career stage (n = 12)***	
Early-career (< 5 years)	0 (0.0)
Mid-career (5–15 years)	6 (50.0)
Senior-career (15 + years)	6 (50.0)
***Academic Discipline (n = 12)***	
Rehabilitation	3 (25.0)
Population and public health	3 (25.0)
Nursing	3 (25.0)
Social work	1 (8.3)
Sociology and criminology	1 (8.3)
Psychiatry	1 (8.3)
***Knowledge user role/lens*** * (n = 8)	
Healthcare professional	4 (50.0)
Person with lived experience of a health condition	1 (8.3)
Health system decision or policy maker	1 (8.3)
Health professional organization representative	1 (8.3)
Manager or administrator	1 (8.3)
Community organization representative	1 (8.3)
Community representative	1 (8.3)

*Cell counts do not add to 8 as some participants identified with more than one role. Unlike self-identified knowledge user roles in the survey,* knowledge user lenses for duals were inferred by the lead author based on their descriptions of professional and community roles during the interview. These lenses are provided for contextual background and are not intended to imply a fixed social identity.*

### Approach to reporting findings

We present synthesized themes drawn from participants’ accounts, using illustrative quotes to highlight key insights. Qualitative descriptors (e.g., a few, some, most) reflect interpretive judgments about salience, not frequency [34]. Differences by role are reported only when they contributed meaningful interpretive depth. Quotes are labelled by participant role (R = researcher, KU = knowledge user, D = dual role) and a numeric identifier, based on interview order. Some identifiers appear non-sequential due to reclassification (e.g., researchers re-coded as duals) and exclusion of one pilot interview.

### Interview themes

We identified three overarching themes: (1) diverse drivers: role-based motivations and perceived effects; (2) It’s all about the relationships; and (2) navigating evolutions and tensions.

## Theme 1: diverse drivers – role-based motivation and perceived effects

There were distinct motivations for working in partnership that varied according to participants’ roles. Role-based distinctions influenced the perceived effects of engaging research partnerships, aligning closely with the participants’ motivations.

### Among researchers: the desire for relevant, impactful research

Researchers were primarily motivated by a desire to produce relevant, meaningful, and impactful research. This motivation was shaped by a blend of personal experiences and broader contextual influences, reflecting their diverse backgrounds. Pivotal experiences often played a role, such as one researcher’s realization during their work in government of the disconnect between science and practice:

*“I worked in government for many years and I realized that the science that I was focused on wasn’t the problem. The problem was that we weren’t giving adequate voice to the people who were concerned about what was happening in their communities and enabling them to engage better with what we know. There was just a real disconnect there.”* (R09).

The diverse backgrounds of researchers provided unique insights and priorities, driving a commitment to research that addresses real-world challenges. The availability of funding initiatives with partnership requirements further fueled interest in partnership-based approaches. Over time, researchers noted an evolution in their approach, recognizing partnered approaches as crucial to their work. While external mandates supported partnership approaches, the commitment to partnership-based approaches was rooted in a genuine belief in their capacity for meaningful change.

#### Seeing tangible impact

Researchers found engaging in research partnerships to be personally enriching, expanding their worldview and contributing to their personal growth, *“It’s also involved with me*,* at least*,* a much greater and in-depth understanding of different ways of looking at research and looking at the world”* (R10). Researchers also expressed that feeling more accountable to end-users led to intentional research practices and a willingness to embrace diverse perspectives. The most significant value, however, came from seeing their research have a tangible impact:

*“For me some of the most meaningful impact is meeting people that used our product*,* in like*,* the weirdest places. Like*,* watching hockey games and talking to a complete stranger and then them figuring out*,* ‘oh my god*,* I’ve actually used something you’ve developed. It was really helpful.’ Stories like that. And it’s happened*,* actually*,* so many times. For them to share that*,* to remember that they’ve used it*,* that speaks to me.” (R02)*.

### Among knowledge users: “it just makes things more human” - enhanced advocacy and credibility

In contrast, knowledge users were motivated by enhanced advocacy, cross-learning, and credibility. Research partnerships were viewed as a strategic avenue to enhance credibility and strengthen advocacy efforts. As one participant noted: *“It gives us credibility also being able to say what we – or cite what we say*,* whatever that expression is. But being able to cite what we’re advocating for and not just relying on research done by others.”* (KU01) This highlights the reciprocal benefits of research partnerships for knowledge users, presenting opportunities not only to contribute their insights, but also to strengthen their authority and influence through evidence-based advocacy, whether in policy discussions, resource allocation, or community initiatives. This was particularly important for knowledge users advocating for underserved communities or underrepresented perspectives, as research partnerships bolstered their authority and ensured that research reflected the lived experiences of those they represent.

Knowledge users recognized the instrumental value of research partnerships, noting how they accelerated processes of change and humanized the research process. As one knowledge user explained:

*“Having patient partners at the table just brings a very unique voice. It just makes things more human. It’s not like*,* ‘Oh*,* we’re just doing this to win a grant*,*’ like*,* ‘We’re doing this because we actually want to change people’s lives.’ And to me*,* that’s incredibly amazing.”* (KU04).

### Among duals: enhancing practical relevance and improving health outcomes

For duals, enhancing practical relevance and improving health outcomes were central motivators. Engaging in research partnerships was not only ingrained and inherent but also viewed as a practical necessity. All duals emphasized the importance of incorporating diverse perspectives in research, recognizing that teamwork enriches dialogue and enhances research quality. They believed that each perspective complements the other, contributing to a more comprehensive understanding and better outcomes:

*“I don’t know that I’d want to do research without a partnership anymore. I think that no one person in any role can know everything. So*,* I can bring my perspective as a mom or a [healthcare provider] or a researcher but I can’t represent any of those uniquely. I bring all of my hats. So any one of those perspectives is affected by the other. And the more people you can have with different perspectives on the team*,* I think it’s just a richer project you’re going to have.”* (D13).

While many duals felt they had always approached research from multiple perspectives, the evolution of these practices had bolstered their legitimacy and acceptance in health research, minimizing the need to defend their use. Participants perceived this enhanced legitimization as fostering a cultural shift in academia that supports interdisciplinary teamwork, values diverse forms of knowledge, and enhances transparency and accountability.

#### A unique blend

Duals experienced a unique blend of role-based partnership effects. Like researchers, they experienced personal growth and expanded perspectives of communities and health issues. Duals also highlighted tangible effects, such as reducing child apprehension rates and demonstrating cost-effectiveness. Like knowledge users, duals valued partnerships for promoting advocacy and accountability, facilitating more responsive research practices and humanizing the research process. However, duals stood out in their focus on ensuring the sustained impact and practical relevance of their work. Their deep commitment to their communities often extended beyond traditional research roles, demonstrating a distinct perspective and understanding of the complexities of bridging research and practice. This practical approach addressed immediate project goals and ensured long-term impact, extending benefits into everyday practice and fostering examples of positive change: *“I think this has been really important because some do this work intuitively and can’t always label it*,* but they just get it done.”* (D11).

## Theme 2: it’s all about the relationships

When describing partnerships that participants felt worked particularly well, it was evident that success hinged on more than the technical aspects of collaboration – it was fundamentally about the quality of relationships fostered among partners. These relationships not only facilitated effective communication and teamwork but also served as normative principles shaping the partnership’s dynamics, sustainability, and ability to navigate challenges.

### There has to be some chemistry there

Successful research partnerships were grounded in principles that emphasized establishing and maintaining relational quality. This required developing skills not taught through formal training, such as humility, trust-building, and flexibility, that participants had to navigate, fumble through, and cultivate over time. Participants emphasized the importance of investing time to connect, build understanding, and value all contributions, regardless of background or role: *“so they respect my position*,* I respect their position. But you can’t think one trumps the other.”* (D08). Some participants noted that strong relationships often evolved over years, beginning well before a grant and continuing long after.

*“You can’t just go into a community and think that you are approaching it on the basis of an equitable and reciprocal partnership*,* because the relationships aren’t there*,* the trust isn’t there.”* (R10).

*“My advice would be to make sure that you spend a lot of time… and it’s not time wasted*,* making connections and asking questions. The project planning is probably the most important part. Allocate as much time as you can to the pre-work you need to do.” (KU03)*.

Flexibility, openness, and a commitment to continuous learning further strengthened research partnerships. Adapting to changing circumstances and acknowledging mistakes required confidence to pivot and humility, described metaphorically as *“parking your ego”* (D11). A few participants described how relational comfort and trust developed gradually through consistent effort and accountability. As one researcher explained:

*“You won’t be trusted right away*,* which makes it even more important that you are accountable from the beginning… because it’s that accountability that ultimately gives you the trust.” (R10)*.

Another reflected on how building trust also required navigating interpersonal boundaries and shifting relational dynamics, especially early on:

“It all comes back to relationship and communication. I think about it in the beginning because you always have that researcher-participant kind of mentality… what boundaries need to stay in place, what boundaries should come down.” (D13).

Sustainability in partnerships depended on mutual support and reciprocity, with both parties contributing to and supporting each other’s work. In essence, relationships were not incidental to partnership success, they were the foundation. Trust, humility, and reciprocity required ongoing, deliberate effort and shaped how partnerships formed, functioned, and endured. This sense of relational quality underpinned partnerships’ perceived success or failure.

### Unpacking power dynamics: navigating principle and reality

Some participants recognized that fostering strong relationships also meant understanding and navigating the complexities of power-sharing. Many described a shift toward more equitable and authentic interactions as partnerships matured. However, they identified early challenges, such as technical language and acronyms, which initially hindered communication and excluded certain voices. Reflecting on these early barriers, one knowledge user noted their discomfort and the need for a more inclusive approach: *“I would often think to myself like what if I didn’t have that*,* would I even feel comfortable sitting at the table? And I think the answer would be no. I think alot of people would get intimidated by that. But now I see that things are becoming a little bit more formalized.”* (KU04). A few researchers similarly noted that knowledge users, particularly parents, sometimes felt hesitant to express their opinions, especially in early partnership stages. Over time, most participants saw improvements in power-sharing dynamics through trial-and-error, facilitated by deliberate efforts to diversify and expand partnership teams. For example, including a wider range of knowledge users helped balance voices at the table and fostered a more democratic decision-making process.

Despite these improvements, a few participants acknowledged that power dynamics remained fluid and complex. Individual preferences, professional backgrounds, and positional authority influenced each team member’s level of involvement and control, leading to variations in decision-making processes. The persistent tension between the principle of sharing power and the realities of enacting power-sharing in practice was a recurring challenge. As one participant explained:

*“It’s really hard*,* right? Especially when you bring in high-level decision-makers or researchers alongside patient partners or youth. It’s difficult to avoid the natural hierarchies that emerge in those conversations.” (D05)*.

A few participants described the role of experienced researchers in challenging these hierarchies. However, they also acknowledge the influence of academia’s hierarchical structure and unequal access to resources, sometimes hindering genuine power sharing.

*“Yes*,* I think we have to be so careful about the power sharing piece. Ultimately*,* I have the money typically… And so what does that really mean*,* like how do we define that? So we can’t say that we don’t hold more power because we do as the researchers”* (D04).

To address these challenges, a few participants highlighted the importance of setting clear guiding principles, creating safe spaces for dialogue, and ensuring that input for all team members was valued. While this required time and effort, participants emphasized the importance of investing early in power-sharing for the long-term success and sustainability of partnerships.

### Productive tensions: adapting to adversity

Navigating challenges was recognized as a natural aspect of working together. Some participants, especially duals, emphasized the importance of authenticity and direct communication in addressing difficulties directly. Open dialogue and collaborative problem-solving were viewed as opportunities to strengthen relationships, even as tensions arose. Participants described trust not only as foundational but as something tested and reinforced through adversity. When present, trust allowed teams to remain engaged, adapt, and grow from challenges.

*“In some ways*,* it brought us closer. That sounds funny. But in talking over how to deal with that difficult situation it made us discuss other things that probably should have been talked about before but hadn’t. And we all ended up kind of banding together in adversity.” (R10).*

### Adopting a long-term perspective

Some researchers emphasized the importance of adopting a long-term perspective, viewing their careers as ongoing engagement. Rather than focusing solely on individual projects, some researchers highlighted the value of building enduring relationships beyond project-based funding. For example, one participant shared how their approach evolved from forming advisory panels for each grant to cultivating more sustainable, long-term partnerships as their research program matured. Meaningful partnerships, participants noted, depend on reciprocal exchange, where researchers both draw on their partners’ expertise and contribute actively in return. They argued that relationships should not be seen as project-specific but as integral to their broader professional journey, reducing transactional interactions and promoting sustained collaboration.

*"So the purview can’t be - oh for this project we’re going to need to work with these people. We just need to think of*,* oh*,* in my career I need to have contact and relationships with these organizations and find a way to foster that. It’s a two-way street. We just can’t be taking" (R02).*

Additionally, a few participants emphasized the importance of starting small and allowing partnerships to grow organically over time. This incremental approach mirrors the evolution of a research program, where one project builds on the success of previous ones, expanding networks and partnerships to align with evolving priorities. By taking a long-term, holistic view, researchers can foster deeper connections and ensure that partnerships remain meaningful and sustainable.

## Theme 3: navigating evolutions and tensions in research partnerships

Participants unanimously recognized that research partnerships exist within systems that are both evolving and prone to tensions. This principally involved the academic system: while some aspects have adapted to support research partnerships, persistent misalignments remained between academic structures, funding expectations, and the core principles of partnership work. These misalignments presented opportunities and challenges, fostering collaboration while complicating its execution.

### Evolution of research partnerships

Most participants noted a shift in academic culture toward embracing research partnerships, reflecting increased acceptance and integration. However, this progress required adjustments from researchers, as one participant remarked, *“Funders really had to pull some academics to get onside with that.”* (R02) While this shift represents progress in integrating partnership work into academia, it also highlights the ongoing challenges and adjustments required to embrace collaborative research fully.

Most participants also described advancements in partnering, with knowledge users playing increasingly significant roles. As one knowledge user reflected, *“I think the rules of engagement have changed substantially. Knowledge users are more involved in research themselves and as part of the projects. And so we’re not just partners on paper anymore" (KU01).* This formalization of knowledge user engagement and evolving funding requirements were thought to have incentivized collaboration.

*“Requirements have changed in terms of what governing bodies or funding bodies have requested*,* so that maybe enforced certain collaborations more than would have happened before”* (KU03).

### Tensions between academic structures and partnership processes

Despite progress, some participants highlighted the ongoing tensions between academic structures and the processes required for effective partnerships. A key challenge highlighted by most researchers was the lack of recognition for partnership work in tenure and promotion systems. As one participant lamented:

*“What’s interesting is that it’s something in our world of academia that’s basically of no value right now*,* like in the tenure system and stuff. No one seems to care – but I think a lot of people that are good applied researchers have a different value system. But if you’re good at doing applied research*,* you also tend to have a pretty good CV*,* so I think it doesn’t matter.”* (R09).

The disconnect between academic expectations and the realities of partnership work often left researchers feeling underrecognized and undervalued. Another researcher expressed, *“So it’s been an uphill battle. I mean the timelines and metrics of doing partnership research with communities are going to be different. And that’s a good thing not a bad thing. But often the academics themselves are penalized for it”* (R10). This tension necessitated a delicate balance between meeting academic standards and fulfilling responsibilities to knowledge users. As expressed by another participant, “*So it’s like who’s at the forefront*,* who’s getting the accolades because of this? Communities typically do not need first-authored publications in a journal but that’s what we’re after” (D04).* This balancing act was particularly challenging given the time and resource-intensive nature of research partnerships compared to dominant biomedical research paradigms within health research.

Institutional barriers added further complexity to the partnership process. A few Participants recounted instances where they were met with bureaucratic hurdles in reimbursing partners and navigating research ethics processes. For example, one participant recounted an incident where they took a parent to a conference and struggled to convince the university to reimburse their costs, emphasizing the need for institutions to evolve their policies to better support partnership activities. Another participant noted that research ethics processes within academic institutions can be challenging to navigate, particularly in child health, indicating a need for greater clarity and understanding among research ethics boards:

*“So those are some of the hurdles that you know sometimes institutionally our ethics and well-intentioned ways of doing things the right way*,* end up not making sense. Somebody wasn’t thinking you know*,* in both cases.”* (R10).

A few participants also identified tensions related to funding mechanisms and the need to strike a balance. One participant reflected on the cyclical nature of funding, noting the need for symbiotic components to do partnership research. These challenges often required researchers to fill gaps themselves. For example, a few described investing time and effort “pro-bono”, building relationships before formal partnerships existed, and continuing collaborative work beyond the life of a grant, reflecting a strong sense of responsibility and commitment to their partners. Participants cautioned against including partner names on grants without genuine engagement, emphasizing the need for funders to consider the implications of their funding strategies on research partnerships. Furthermore, participants suggested that funding structures may not always align with the principles of genuine partnerships, sometimes leading to compromises in partnership integrity.

*“I think it’s more advice for funders; the funders are putting us in these compromising positions of ultimately – like the funding agencies are really defining everything. And if we are researchers and we’re wanting to get grants and get tenure promotion*,* we play that game. But they’re really putting – and I have experience– put us in compromising positions.”* (D04).

## Discussion

This study advances understanding of partnership dynamics by exploring how child health researchers and knowledge users navigate the partnered health research process. Specifically, we highlight the foundational role of relational quality and the distinct role-based motivations that shape engagement. Importantly, this study also highlights the ongoing structural misalignments between partnership work and academic structures. These tensions reflect the growing pains of integrating partnership work into academia, where traditional norms and expectations often conflict with the time, care, and sustained commitment that effective partnerships require. By deepening our understanding of these dynamics, this study offers insights into how partnerships function, what factors contribute to their success, and how they can be better supported within health research systems. Additionally, it highlights how some individuals navigate these dynamics from dual perspectives, a role that warrants further exploration in partnership-based research.

### The central role of relationships in partnership dynamics

Relationships are widely recognized as important in research partnerships [20, 23, 41–43], and this study reaffirms and expands on their significance, demonstrating that relational quality, characterized by trust, respect, flexibility, and reciprocity, is fundamental to partnership success. These elements consistently recurred in discussions, underscoring their central role in shaping partnership experiences. Although these principles may seem intuitive, they are often undervalued in professional and institutional settings [44]. Notably, these findings align with longstanding Indigenous scholarship on relationality, which has long emphasized relational accountability, mutual respect, and shared responsibility in knowledge creation [45–49]. While health research increasingly acknowledges the value of interpersonal dynamics [20], these values have long been foundational in other knowledge traditions.

The strength of relationships also shaped how challenges were navigated. Participants’ openness about difficulties contrasts with the literature’s tendency to focus on successes [23, 50], and they emphasized addressing difficulties through relational strategies such as open communication, transparency, and a willingness to learn from tensions. This suggests that partnerships rooted in strong connections may be more resilient and adaptable in the face of adversity [43]. Power-sharing was similarly described as a fluid, evolving process that improved as relationships deepened and partners gained experience.

Even so, structural misalignments such as tenure policies, funding structures, and ethics processes remain persistent obstacles to fully supporting partnership work [16, 41]. Broader critiques of academic health institutions similarly note that partnership-based research remains undervalued, lacks adequate infrastructure, or is misaligned with existing incentive structures (51). These critiques call for more relational, power-conscious approaches that recognize and support the interpersonal work required for effective partnerships [51]. This also includes fostering reflexivity among researchers, who play a central role in navigating power dynamics and ensuring partnerships remain responsive and equitable [29]. Addressing these structural barriers requires not only ongoing institutional evolution but intentional efforts within partnerships themselves. Deliberate investment in relationship building, through time, resources, and relational factiliation [20, 52, 53], can help partnerships move beyond transactional or project-based collaboration. Enhancing institutional recognition of this often invisible work [44, 54] is also equally important to support sustained, meaningful engagement.

### Understanding motivations and role dynamics

This study contributes to a small but growing body of literature examining the motivations that shape engagement in research partnerships. Our findings underscore that motivations are not uniform across roles. Researchers often described being driven by a desire to conduct meaningful and impactful research, knowledge users emphasized advocacy and community change, and individuals in dual roles highlighted the importance of practical outcomes. These motivations were often implicit, yet shaped how partners viewed their roles and contributions. This aligns with emerging evidence suggesting that both role-specific and shared motivations underlie partnership work, with discrepancies often emerging early and converging over time through shared goals and relationship-building [19, 21, 55]. When left unspoken, underlying assumptions about partnership goals can contribute to common partnership barriers such as role ambiguity and misaligned expectations [20, 43]. By proactively discussing motivations early, partnerships may be better positioned to clarify roles and expectations (reducing the risk of misalignment), identify shared priorities (strengthening collaboration and research relevance), and sustain engagement (as partners see how their contributions align with their goals).

Our findings also reinforce the importance of nurturing the intrinsic and interpersonal [55] motivators for partnered research [56, 57], aligning with others [58] who suggest that while external motivators (e.g., funding, incentives) play a role, they are not the primary drivers of partnership work and, on their own, are insufficient to sustain engagement. As others have argued [19, 29], revisiting motivations throughout the research process can build trust, clarify evolving goals, and support more resilient and equitable partnerships.

### The opportunity of the dual role

A noteworthy finding of this study is the “dual role,” where some researchers drew on both academic training and practice-based experience, offering a unique vantage point informed by their prior or ongoing work in community, clinical, or policy settings. These participants were uniquely equipped to bridge academic and healthcare environments, demonstrating a nuanced sensitivity to the broader contexts in which research is applied. Their dual perspective often enhanced team development, strengthened the practical relevance of research, and contributed to research processes that were more closely aligned with real-world priorities. However, their dual roles also introduced complexities. Participants described blurred boundaries between their research and applied responsibilities, which could create role strain or ambiguity.

While the concept of dual roles has been acknowledged in the literature using terms such as boundary spanners [59], intermediaries [60], embedded researchers [61], and pracademics [62], its specific contributions to partnered health research remain underexplored. For instance, boundary spanners are recognized as key facilitators of early-stage iKT [63], while roles such as opinion leaders and local champions are recognized as implementation strategies that strengthen collaborative functioning [64]. These overlaps point to a growing intersection between implementation science and partnership-based research [65].

Collins’ concept of the *outsider within* [66] offers a helpful lens to interpret these findings. Originally developed to describe how individuals at the intersection of unequal social systems can offer critical insight from within, this framing helps illuminate how dual-role individuals appear to draw on multiple standpoints to recognize tensions, bridge gaps, and foster connections across institutional and professional boundaries. Their ability to perceive and navigate dynamics that may be invisible to others constituted a form of situated expertise that shaped how they contributed to partnerships. Given global investments in embedded researchers [61, 67, 68], further exploring how this positioning contributes to partnered health research may be important.

### Implications for practice

Findings from this study underscore the importance of attending to the relational foundations of partnered research. Establishing trust, fostering reciprocal engagement, and navigating role-specific motivations require time, humility, and adaptability – capacities that have often been underemphasized or insufficiently supported in academic environments. Encouragingly, national initiatives such as CIHR’s pan-Canadian Training Modernization Strategy signal a shift toward more systematically embedding relational and collaborative competencies into doctoral training in Canadian institutions [69]. This framework emphasizes inclusive, culturally safe engagement, cross-sector collaboration, and interpersonal capacities such as humility, trust, and reflexivity, humility, signaling a meaningful shift in how these competencies are recognized and nurtured.

Findings also point to the need to better bridge the structural divisions between academic and applied spheres. Participants who occupied roles across systems offered particularly nuanced insights. Yet current systems often reinforce binary distinctions between researchers and knowledge users, which can limit formal recognition and support of those operating across these boundaries. Academic and health system leaders should continue to consider ways to embed, train, and support these individuals, including through dedicated roles, flexible career pathways, and evaluation criteria that reflect collaborative and cross-sector work.

Pragmatic steps at the project level include allocating time and resources for relationship-building during project planning [70–72], creating mechanisms for meaningful engagement beyond advisory roles (e.g., shared leadership positions), and co-developing clear goals and expectations with partners, with a commitment to revisiting them over time. Institutions and funders can further support this work by embedding relational competencies (e.g., communication, reflexivity, implicit bias training) into research training, evaluation criteria, and funding guidelines.

### Study limitations

Despite efforts to recruit early-career researchers, the sample consisted exclusively of mid- and late-career researchers. Their perspectives, shaped by years of experience, institutional knowledge, and established networks, offered valuable insights on the practical realities of partnership work. However, the absence of early-career researchers limits understanding of how factors such as power-sharing, academic expectations, and the substantial time commitments required for partnership work are navigated early in a research career. Including early-career perspectives in future research would provide a more comprehensive understanding of how engagement evolves and is shaped across career stages.

Additionally, only three knowledge users participated, limiting the diversity of perspectives from this group. While this represented a higher proportional response rate than that of eligible researchers, it does not reflect the range of knowledge user experiences. This imbalance reflects both the composition of our recruitment pool, drawn from a national survey of partnered child health research projects [38], and broader patterns in health research, where researchers often outnumber knowledge users in funded partnerships. While we analyzed responses by role and identified both convergence and divergence, the findings predominantly reflect researcher perspectives and should be interpreted with that in mind.

Furthermore, the perspectives of children and youth were not captured. All knowledge users were adults acting in professional roles on behalf of children. As such, the findings reflect adult views on partnership work and do not represent the lived experiences or engagement preferences of children and youth.

Our definition of dual-role participants was limited to those with formal academic appointments who also held health system or clinical roles. While this allowed us to explore how institutional affiliations shape boundary-spanning experiences, it may not capture the full spectrum of intersecting identities held by others in the sample, such as those with lived experience, caregiving responsibilities, or professional backgrounds outside traditional academic or clinical roles.

Finally, while this study was situated in child health, many of the experiences and dynamics described by participants may be broadly relevant across other areas of health research. A forthcoming mixed-methods study explores whether and how the child health context shapes research partnerships in distinct ways.

### Implications for future research

Future studies should explore partnership dynamics among early-career researchers, whose institutional precarity, and limited discretionary time may present unique barriers to partnership work. Understanding how power-sharing ideals intersect with the practical time commitments and academic expectations at early career stages could help identify tailored supports to foster meaningful engagement. Additionally, more research is needed to explore the evolving roles and contributions of knowledge users, particularly those from underrepresented groups (e.g., children and youth) in child health. Comparative studies across health domains could deepen understanding of how contextual factors shape partnership dynamics and inform transferable lessons across settings. Further examination of dual-role individuals may also provide insight into how cross-role perspectives facilitate or complicate co-production and implementation. While our findings point to the value of these experiences, the concept of duality itself warrants further exploration. Future research could help refine and expand this idea by examining diverse forms of dual positioning, including roles shaped by lived experience, community leadership, or professional transitions, and the supports and constraints these individuals encounter across systems. A broader conceptualization may help surface additional forms of cross-role contributions that are not formally recognized, and embracing this complexity could add richness and depth to both the dynamics and outcomes of research partnerships.

## Conclusion

This study advances our understanding of the role-based variations in research partnerships by unpacking the distinct yet synergistic motivation and effects among researchers, knowledge users, and individuals with dual roles. The findings reaffirm the central importance of relationships in navigating and sustaining research partnerships and highlight the continued need for academic institutions and funders to adapt their structures and policies to better support partnership-based research. At the same time, researchers have a role in championing and demonstrating the value of partnership work to help drive systemic change. Further research is needed to explore partnered child health research’s unique complexities and deepen our understanding of dual roles in facilitating research partnerships.

## Supplementary Information

Below is the link to the electronic supplementary material.

Supplementary Material 1

Supplementary Material 2

## Data Availability

No datasets were generated or analysed during the current study.
