# Supplementary material for: ‘There has to be some chemistry there’: an interpretive description exploring the experiences, motivations and dynamics of partnered child health research
Source: Res Involv Engagem. 2025 Aug 29;11:105. doi: 10.1186/s40900-025-00777-1 (PMC12395850; doi:10.1186/s40900-025-00777-1)
Supplement: Supplementary file 1 — Supplementary Material 1 [file 40900_2025_777_MOESM1_ESM.docx]

Supplementary file 1

GRIPP2 Short Form – “There has to be some chemistry there”: an interpretive description exploring the experiences, motivations, and dynamics of partnered child health research

| Section and topic | Item |
| --- | --- |
| 1: Aim  Report the aim of the study | The study aimed to understand the experiences and lived perspectives of researchers and knowledge users engaged in partnered child health research. PPI was included to support reflexivity and provide additional perspectives during analysis and interpretation. |
| 2: Methods  Provide a clear description of the methods used for PPI in the study | An interprofessional colleague with lived experience of a congenital health condition was involved during the data analysis, interpretation, and dissemination phases. Based on their preferred involvement, they independently reviewed two transcripts, contributed to coding discussions, and offered contextual insights grounded in personal experiences with healthcare and health research. Their contributions were considered on equal footing with those of other co-authors. They also reviewed and provided feedback on manuscript drafts. Engagement occurred through virtual meetings and email. |
| 3: Study results  Outcomes – report the results of PPI in the study, including both positive and negative outcomes | Our team member with lived experience contributed an alternative lens during data analysis, which enriched interpretation of findings and helped shape how results were framed and communicated. Their reflections informed how themes were refined. However, because the patient partner joined during the project analysis phase, they were not involved in shaping the study design or developing the data collection tools. |
| 4: Discussion and conclusions  Outcomes – comment on the extent to which PPI influenced the overall study. Describe positive and negative effects | PPI added interpretive value during analysis and strengthened the relevance and clarity of reported findings. KCL’s contextual insight informed theme refinement and the language used in dissemination. Earlier involvement may have supported more comprehensive contributions across study phases, including study conceptualization. |
| 5. Reflections/critical perspective  Comment critically on the study, reflecting on the things that went well and those that did not, so that others can learn from this experience | Involving a person with lived experience of a congenital health condition during analysis supported reflexivity and deeper consideration of how findings were interpreted. Their independent review of transcripts and personal reflections prompted the lead researcher to critically examine coding decisions and language choices. While involvement was limited to later stages, it was beneficial. Future studies should plan for earlier and broader engagement to support meaningful contributions across all research stages. |

Table 1. GRIPP2-SF checklist for the manuscript “There has to be some chemistry there”: an interpretive description exploring the experiences, motivations, and dynamics of partnered child health research

Reference:

1. Staniszewska, S., Brett, J., Simera, I. *et al.* GRIPP2 reporting checklists: tools to improve reporting of patient and public involvement in research. *Res Involv Engagem* **3**, 13 (2017). https://doi.org/10.1186/s40900-017-0062-2
