# Supplementary material for: ‘There has to be some chemistry there’: an interpretive description exploring the experiences, motivations and dynamics of partnered child health research
Source: Res Involv Engagem. 2025 Aug 29;11:105. doi: 10.1186/s40900-025-00777-1 (PMC12395850; doi:10.1186/s40900-025-00777-1)
Supplement: Supplementary file 2 — Supplementary Material 2 [file 40900_2025_777_MOESM2_ESM.docx]

Supplementary file 1

INTERVIEW GUIDE

Opening – briefly explain:

- Study purpose and who I am
- Why participants were contacted (responded to a survey in summer 2020 regarding experiences in “X” project grant); interested today in experiences overall
- This study has been approved by the University of Manitoba Health Research Ethics board. Please note that your responses will be kept confidential and that you don’t have to answer any questions that you don’t want to. Given that you also responded to the survey, your answers today may be linked to the survey for greater understanding.
- *Before I start, do you have any questions?*
- *I will be audio recording our interview to allow for thorough documentation. All interviews will be de-identified for reporting purposes, kept on a password protected server, and destroyed after 10 years. Do I have your consent to proceed with the interview and audio recording of this interview?*

1. To start, can you first tell me a little bit about yourself? (eg. background, training environment (if applicable), research interests)

1. What was it about your research journey that led you to be involved in partnership style research?

1. For how long would you say you have been doing partnership style research? When thinking broadly about all of the different research partnerships you’ve been involved in over X years, what comes to mind?

1. For those with lots of experience with research partnerships:

- Can you reflect on how the various partnerships you have been involved in have worked over time?
- Which of your experiences in working in partnership do you feel worked best?

Probes: explore what made it so successful in their view? Can you think of a partnership where maybe that ideal wasn’t achieved and why?

  For those with few partnership experiences:

- Can you tell me a little bit about your most recent partnership?
- Do you have an experience with a different partnership that was perhaps managed differently?

Additional potential probes:

- *Tell me about your experience working with knowledge users (or for knowledge users, working with researchers)*
- *Who initiated the partnership, how did you become involved?*
- *Why did you get involved? What incentives, if anything, motivated you?*
- *Where there any strategies that worked well/didn’t work well*
- *Not all people want to be engaged at the same level. How did you navigate that as a team?*
- *How were decisions made within the team? Were other aspects of power shared within the team? (eg. how project resources were allocated, how priorities for the research were established, etc).*
- *How do you think the opinions and expertise of various partners have been included in the projects you’ve been involved in (eg. decision-making, co-production)*

1. When thinking about all of these experiences, what would be an ideal partnership?

- How does this compare to your actual experience?
- *Probe: What factors do you attribute to this? What factors or attributes are needed for successful partnership research?*

1. As I mentioned, I’m studying partnership research in child health for this project. Do you have experience in partnership research focused on other populations (eg. adults)?

- *If yes*, how do you think the two might have been different based on your experience?
- *If no*, why have you chosen to focus on child health? Is there anything you’ve learned over time that you feel has influenced your ability to engage?
- Probe about engaging with various knowledge users in child health (i.e, children and youth, parents, others)
- Have there been any special considerations you’ve had to make because your partnerships was focused on child health?

1. What value do you feel a partnership approach has brought to the projects you’ve been involved in?

- Probes: personal, research, health or health policy outcomes
- What was achieved through working in partnership? (probe: what would have been different if a partnership approach was not used?)
- Have there been any challenging or negative outcomes that have come from the projects you’ve been engaged in?

1. Moving forward, do you intend to engage in other health research partnerships?

- Why or why not?

1. Based on the experiences you’ve shared with me today, do you have any words of advice that you would bring forward into your next partnership?

1. That’s all the questions I have for you today. Based on what we talked about, is there anything I’ve missed or anything else you’d like to share?

Closing: Thank participants for their time
